# Nanoscopic oxygen control of functional oxide nanoparticles by electro-chemical route at ambient temperature

**DOI:** 10.1186/s11671-024-03969-y

**Published:** 2024-02-08

**Authors:** Putul Malla Chowdhury, A. K. Raychaudhuri

**Affiliations:** 1 Department of Physics, Netaji Nagar College for Women, 170/13/1, Netaji Subhas Chandra Bose Road, Regent Estate, Kolkata, 700092 India; 2 Central Glass and Ceramic Research Institute, 196, Raja Subodh Chandra Mallick Rd, Jadavpur, Kolkata, 700032 India

**Keywords:** Perovskite nanoparticle, Electrochemical oxidation, Oxygen stoichiometry

## Abstract

La_0.67_Ca_0.33_MnO_δ_ nanoparticles of approximate size ∼ 4 nm have been prepared by the chemical solution deposition method to investigate effect of oxygen stoichiometry in the nanoparticles without changing their sizes. Electrochemical oxidation method has been used to change the oxygen stoichiometry $\delta$ at room temperature, which unlike conventional methods to change oxygen stoichiometry by heating in controlled ambience, does not lead to any significant change in size. This has allowed us to investigate the effects of stoichiometry variations in the nanoparticles with no change in size. The unit cell volume, lattice constants and orthorhombic strains of the as prepared sample (with $\delta$ = 2.74) are changed by incorporation of oxygen by electrochemical oxidation which in turns affects the magnetic properties. In addition, oxidation leads to change in oxygen stoichiometry of the magnetically “dead” surface layer on the nanoparticles which also affects their magnetization and coercive field.

**Supplementary Information:**

The online version contains supplementary material available at 10.1186/s11671-024-03969-y.

## Introduction

The physical properties of oxides like perovskite oxides can be controlled by changing the oxygen content which is commonly referred as “oxygen stoichiometry”. In the bulks and films of different perovskite oxides, the effect of oxygen nonstoichiometry has been studied rigorously for a long time [1–4]. There are also investigations of effect of oxygen content on the magnetic properties. For instance in case of manganites, the change in oxygen content brings about a change in its magnetic ordering [5, 6]. This has also been seen in other materials as well [7, 8]. The most common route to change oxygen stoichiometry is heat treatment (typically > 500 °C) in an ambience of controlled oxygen partial pressure [1–4, 9]. Often, for depleting oxygen, the heat treatment is done in a reducing atmosphere. Such heat treatment is routine for bulks and films, as well as micron-sized powders, but it poses a serious problem when used with nanoparticles, particularly nanoparticles with a diameter of < 10 nm. The heat treatment not only changes the oxygen stoichiometry, it also concomitantly enhances the particle size, mainly by agglomeration. Because of similar impacts on physical properties, the effects of change in particle size and oxygen content variations can often not be clearly separated. This necessitates an athermal method that does not need heat treatment at elevated temperature so that control of the Oxygen stoichiometry can be done without size change.

Electrochemical oxidation/reductionis a tool to change the oxygen stoichiometry at ambient condition [10, 11] particularly for nanoparticles. Previously electrochemical method has been used to change oxygen stoichiometry in Ruddlesden-Popper phase oxides like Lu_2_CuO_4_ [12, 13], La_4_Ni_3_O_10_ [14], La_2_CuO_4_ [15], SmBaCo_2−x_Mn_x_O_5+δ_ [16] and double perovskites like BaPrMn_1.75_Co_0.25_O_5+δ_ nano sheets [17]. There are also reports of use of this method for stoichiometry control infinite layer oxides like La_0.2_Sr_0.8_CoO_3-δ_ [18]. However, the particle size used in the past studies is typically of diameter greater than 10 nm. Control of oxygen stoichiometry by oxidation/reduction to control properties of oxide thin films have been used to make thin film Field Effect Transistors with electrolyte or aqueous gates [19–22].

In this investigation we used electrochemical method to have a nanoscopic modification of oxygen stoichiometry at ambient condition in a perovskite oxide manganite nanoparticles with average particle diameter < 5 nm. The structural, magnetic and transport properties of nanoparticles of perovskite oxide manganites have critical dependence on both, size as well as oxygen stoichiometry. The oxygen stoichiometry control as well as substitution with divalent cations at A-site in manganites leads to hole doping which in turn leads to important changes in physical properties which are often accompanied by important structural changes [23]. The enabling role of oxygen stoichiometry in LaMnO_3+δ_ has been investigated and the critical role of $\delta$ has been established in bulk manganites [24, 25], as well as in nanoparticles (diameter ~ 40 nm) [26]. The antiferromagnetic LaMnO_3_ develops a ferromagnetic order for a small change in oxygen stoichiometry (that leads to hole doping) becoming ferromagnetic for $\delta \ge 0.1$. Neutron investigations in nanoparticles of LaMnO_3+δ_ ($\delta \approx 0.03$) showed that size reduction leads to weakening of the Jahn–Teller distortion leading to canting of spin sublattice which destabilizes the AFM order [26]. Thus in nanoparticles of manganites the issues of stoichiometry as well as size reduction are related due to strong coupling of spin, lattice and change degree of freedoms.

In substituted manganites it has been shown that size reduction below 50 nm stabilizes the ferromagnetic phase. In half doped manganites that show charge ordering and strong lattice distortion at the onset of the charge ordering, the size reduction suppresses the lattice distortion and mitigates charge ordering [27]. Extensive investigation on optimally doped La_0.67_Ca_0.33_MnO_3_ nanoparticles have shown that the paramagnetic insulating (PI) to ferromagnetic metal (FM) transition temperature (*T*_C_) varies non-monotonically in the nanomanganite due to the decrease of particle size from bulk to 15 nm [28]. The *T*_C_ is initially enhanced as the size is reduced and it shows a maximum for a nanoparticle diameter $d\approx 50 nm$ and then it decreases again along with suppression of the saturation magnetization ${M}_{S}$. It was found that these changes are linked to the bond angle Mn–O–Mn as well as bond length Mn–O [28].

In this report we carry out an investigation of the effect of variation of oxygen stoichiometry in La_0.67_Ca_0.33_MnO_δ_ nanoparticles with average diameter in the range of below 5 nm, done in a way that there is no change in size of the nanoparticles during the oxidation/reduction process. This investigation also explores ferromagnetism in such small nanoparticles since; the existence of ferromagnetism in La_0.67_Ca_0.33_MnO_δ_ (LCMO) nanoparticles with size below 10 nm has not been investigated before. The present investigation also involves structural analysis to explore how the stoichiometry affects the structure and such properties as variation of transition temperature *T*_C_, the saturation magnetization (${M}_{S}$) and the Coercive field (*H*_C_). As noted before, the effect of variation of $\delta$ in such small nanoparticles could not be carried out because conventional heating method cannot be employed. In this report we used the enabling technique of electrochemical oxidation/reduction as explained before which allows performance of such critical experiments and permit us to change $\delta$ in the range 2.74–3.2 without changing the average diameter of the nanoparticles that stay below 5 nm.

## Method

### Preparation of nanoparticles

La_0.67_Ca_0.33_MnO_δ_ nanoparticles were prepared by chemical solution deposition method (CSD) [29]. In CSD method high purity Lanthanum acetate hydrate, Calcium acetate hydrate and Manganese acetate tetrahydrate were mixed with de-ionized (DI) water and Acetic acid in stoichiometric ratio. Appropriate amount of ethylene glycol were added. The polymer (ethylene glycol) was used forming a network of cations which assisted the reaction in phase formation. The solution was heated and stirred at 70 °C for making it in gel formation. The gel was dried for 15 hours at temperature ~ 150 °C. After that pyrolysis process was done at 350 °C and 450 °C. Then finally it was sintered at 650 °C for 3 hours to obtain the proper phase and the desired particle size. The as prepared particles are referred to as S_1_.

### ***Change of oxygen content (***${\varvec{\delta}}$***) by controlled electrochemical oxidation/reduction:***

**Fig. 1:**
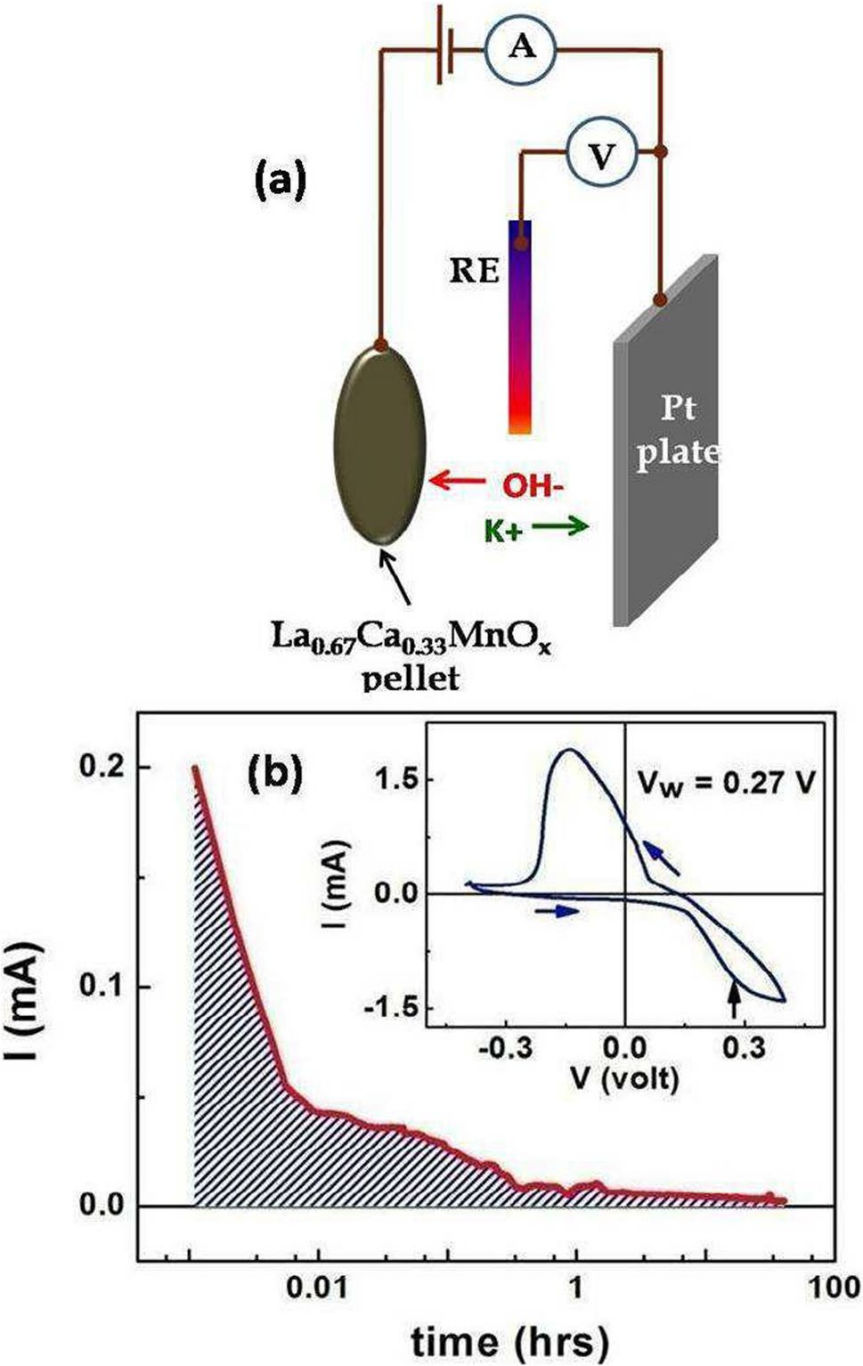
**a** Schematic diagram of electrochemical oxidation process. **b** The current versus time profile during oxidation. Area under the curve is indicated by shadow lines. The inset shows a typical cyclic voltammetry curve. Arrow indicates the working potential (*V*_*W*_) for oxidation

The oxygen content was changed by electrochemical oxidation/reduction method at ambient temperature (∼ 300 K). A schematic diagram of electrochemical process is given in Fig. 1a. We used a conventional three electrode set-up in which the sample was used as working electrode in the form of a thin disk shape pellet. A rectangular thin Platinum sheet and Hg/HgO were used as counter electrode and reference electrode respectively with 1(M) KOH solution was used as electrolyte. Electrical lead and the contact areas were covered by Polymethyl Methyl Acrylate (PMMA) to avoid any spurious charge deposition at the time of electrochemical process. Fig. 1b shows the current vs. time (*I* $-$ *t*) profile of the oxidation process. The total amount of deposited charge $Q \left({t}_{tot}\right)$ on the working electrode (i.e., the sample) can be measured by calculating the area under the *I* $-$ *t* curve by integrating the current $I$ so that $Q \left({t}_{tot}\right)={\int }_{0}^{{t}_{tot}}Idt$, where ${t}_{tot}$ is the total time of deposition. In this case, the Oxygen ions constitute the current. To change the oxygen stoichiometry in manganite films at room temperature previously we have also used this electrochemical oxidation technique [30, 31].

Inset of Fig. 1b shows the Cyclic Voltametry curve (*I* $-$ *V*) for a complete cycle. This shows the potentials for most efficient oxidation (positive axis) and reduction (negative axis) process. From the Cyclic Voltametry curve we fixed the potential to be used for oxidation (*V*_*W*_) at 0.27 V and that for reduction (*V*_*R*_) at $-$ 0.15 V. The width of the pellets is in the range of 0.6–1.0 mm. We have oxidized both sides of the pellets. For homogeneous oxygenation we repeated the process at least 4 times by crushing the pellet after the process, palletizing and then repeating the electrochemical process.

### Sample characterization

The crystallographic phase of the LCMO nanoparticles made by CSD method was confirmed by X-ray diffraction (XRD) technique and the composition was checked by Energy dispersive X-ray (EDX) spectroscopy. The particle sizes were measured by High Resolution Transmission Electron Microscope (HRTEM) images. Magnetization measurements were done in vibrating sample magnetometer (VSM) from 80 to 350 K with field range up to 1.6 T.

### ***Quantitatively fixing the value of ***${\varvec{\delta}}$

One of the crucial parameters that needs be fixed in this experiment is the oxygen stoichiometry $\delta$ of the LCMO nanoparticles. The efficacy of the electrochemical process (oxidation/reduction) can be quantified by proper fixing of the parameter $\delta$. This was done using three different methods described below. The incremental change in $\delta$ can be directly estimated from the Faraday Law of electrochemical deposition. The change in $\delta$ for the total deposition time ${t}_{tot}$ is given as:

1 $${\delta }_{f}-{\delta }_{i}= \frac{Q({t}_{tot} )M}{nem{N}_{A}}$$

where, ${\delta }_{f}$ and ${\delta }_{i}$ are the final and initial stoichiometries, $Q\left({t}_{tot}\right)$ is the total deposited charge in the electrochemical process as defined before. Molecular weight and mass of the sample are *M* and *m* respectively. Avogadro’s number is *N*_A_, *e* = 1.6 × 10^−19^ C is the electron charge and *n* is the valency of the Oxygen ion which is = 2.

While the changes in $\delta$ can be measured precisely from the Faraday Law, the determination of the absolute value of $\delta$ needs other techniques. The value of $\delta$ was measured independently using Iodometric titration and Rietveld refinement of XRD powder patterns. To analyze the crystal structures and for finding out the value of $\delta$, we used Rietveld refinement method for profile fitting of the XRD data using orthorhombic Pnma space group. The XRD data and the results of the refinements are shown in the next section.

For the as grown sample S_1_ the initial value of ${\delta }_{i}$ was determined from the iodometric titration method. For other samples that were oxidized /reduced electrochemically the incremental values of $\Delta \delta$ (= ${\delta }_{f}-{\delta }_{i}$) were determined from the Farday Law. The $\delta$ values of all the samples used are shown in Table 1. The agreement of the values of $\delta$ measured by three methods within their stated accuracy is very good.

**Table 1:** Sample ID and oxygen stoichiometry $\delta$ as determined by three methods along with the average diameter determined by TEM

Sample-ID	$\delta$ (Rietveld refinement)	$\delta$ (Faraday’s Law)	$\delta$ (Iodometric titration)	< d > Average particle diameter (nm)*
S_1_	2.74	2.74	2.74	4.0
*Oxidized samples*				
S_2_	2.76	2.76	-	3.6
S_3_	2.78	2.79	-	3.7
S_4_	2.90	2.91	-	3.5
S_5_	3.20	3.23	3.22	4.0
*Reduced sample*				
S^⋆^	3.14	3.14	3.14	3.7

*S*_*1*_: As prepared sample, *S*_*2*_*-S*_*5*_: S_1_ progressively oxidized electrochemically, S^*^: S_5_ reduced subsequently

*The uncertainties in < d > are approximately ± 1 nm

## Results

### Crystal structure and the particle size

**Fig. 2:**
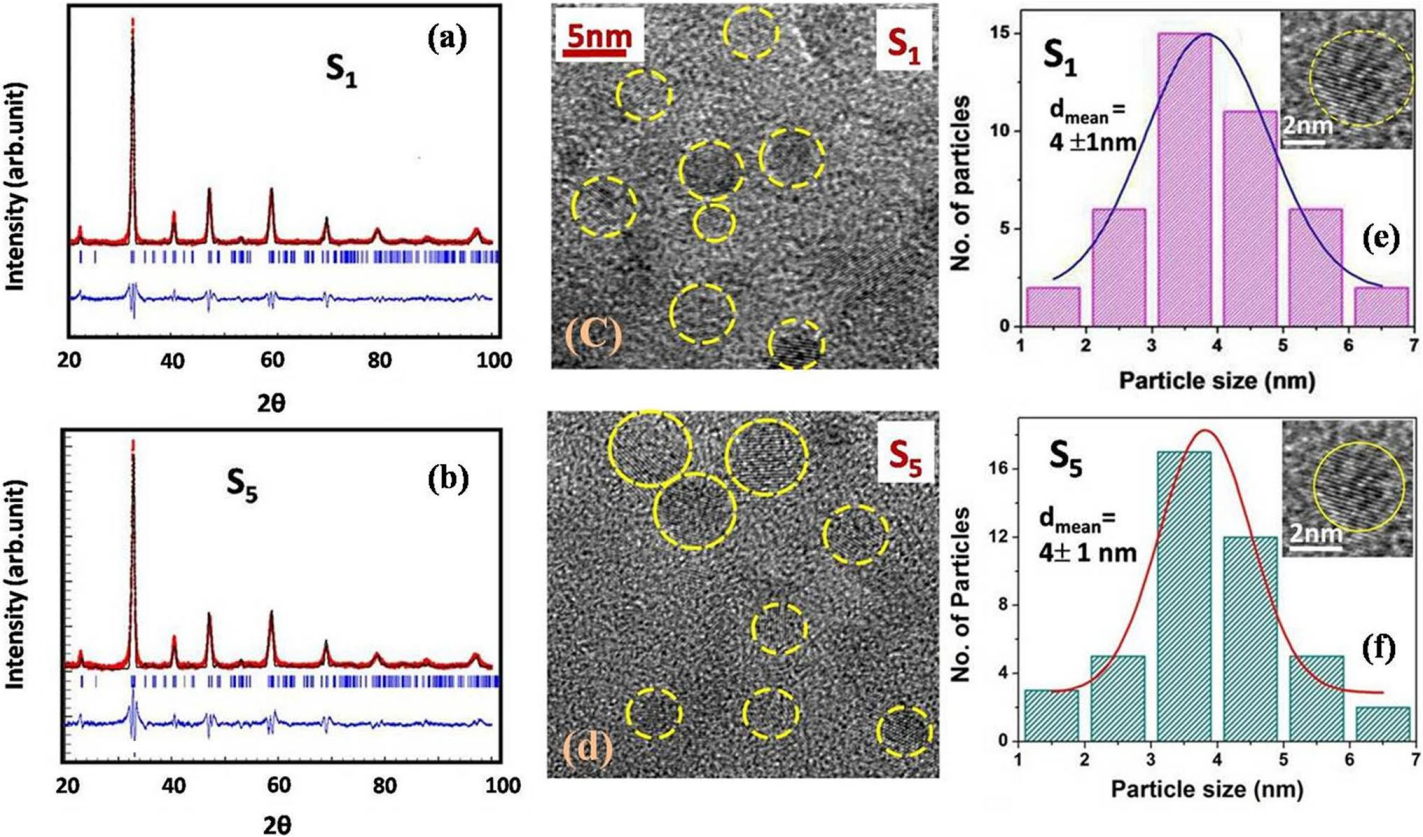
The XRD Rietveld profile fit with residues at 300 K **a** of sample S_1_ (*δ* = 2.74) and **b** of sample S_5_ (*δ* = 3.20). **c** and **d** show TEM image of particles S_1_ and S_5_ respectively. **e** and **f** show particle size distribution of S_1_ and S_5_ respectively. Single particle TEM images of S_1_ and S_5_ are shown in the insets of **e** and **f**

Fig. 2a and b show the XRD patterns (red lines), the Rietveld refined peaks (black lines) and residues (blue lines) for samples S_1_ and S_5_ respectively. In Fig. 2c and d we show the TEM images for the same samples S_1_ and S_5_. Fig. 2e and f show the particle size distribution of S_1_ and S_5_. The insets of Fig. 2e and f show TEM images of a single nanoparticle S_1_ and S_5_.

The size distributions of the nanoparticles were checked after each process of electrochemical oxidation and reduction to ascertain that the average size of the particles have not changed by the electrochemical process. In Table 1 the average particle diameters $<d>$ of all the oxidized and reduced samples are given. Table 1 shows that after the electrochemical process, within the standard uncertainty (± 1 nm), the average particle size remains unchanged. This particular check is important because it validates the claim that the electrochemical process can effectively change $\delta$ without changing the size.

We note that the average particle size in this investigation has been measured by using TEM images that could ascertain size distribution and the average particle size has been determined from this distribution. Global probe like XRD can also be used for particle size determination but we note the limitations that such a probe has for the size range used in this work. Often use of XRD line width and use of such methods like Williamson-Hall (WH) plot from XRD lead to somewhat larger average particle size compared to that found from TEM analysis primarily due to agglomeration of smaller particles to larger particles (see supplementary section).

The values of the oxygen stoichiometry $\delta$ have been determined by different methods mainly to check that for a given sample the values of $\delta$ are internally consistent. The error for the different methods is different due to the nature of measurements. The error bar on the incremental change of value of $\delta$ as obtained directly From Faraday’s Law is the least and is $\approx \pm$ 0.002. This least as it depends on measurement of current and time. This error bar is unchanged from sample to sample. The next higher error bar is in Titration (a volumetric method), where the error $\approx \pm$ 0.021 depends on the accuracy of the apparatus and the accuracy with which the end point is determined. The error bar when one considers sample to sample increase by a factor of 2 at most. The largest error bar in determination of $\delta \approx \pm 0.035$ occurs for the Rietveld method which is large as it is refinement of profile fitting of all the picks. This is done by taking different sample volumes and thus the sample to sample variation is of the same order. In view of the above discussion, we put a conservative estimate on the absolute value of $\delta \approx \pm 0.04$. It is also noted that for the incremental change in $\delta$ as measured by Fraraday’s Law stays at $\delta \approx \pm$ 0.002.

Crystal structure analysis has been done from x-ray diffraction pattern by Rietveld refinement method as stated earlier. In Table 2 the lattice parameters (*a*, *b* and *c*) are compared with those of the bulk and those of the sample with 15 nm average diameter particle. Results show that all lattice parameters (except *c*) and unit cell volume (*V*) are largest for particle with diameter 15 nm, whereas, *c* maximum for the sample with lowest diameter (∼ 4 nm). This implies that the unit cell is extended along the c-axis which enhances the Orthorhombic distortion strain characterized by the following parameters: orthorhombic strain in ac-plane ${OS}_{\parallel }$ and strain along the b-axis with respect to ac-plane ${OS}_{\perp }$. These parameters are defined as:

**Table 2:** Comparison in the structural parameters of the bulk and the nanoparticle samples with average size 15 nm and 4 nm

Structural parameters	Bulk ∗	Nanoparticle (diameter ∼15 nm ∗)	Nanoparticle (diameter ∼4 nm)
a	5.433 A°	5.433 A°	5.429 A°
b	7.687 A°	7.707 A°	7.664 A°
c	5.473 A°	5.473 A°	5.506 A°
V	228.33 (A°)^3^	229.50 (A°)^3^	229.12 (A^0^)^3^
OS_‖_	5.5 × 10^−3^	6.2 × 10^−3^	14.0 × 10^−3^
OS_⊥_	2.0 × 10^−3^	0.1 × 10^−3^	8.9 × 10^−3^
Mn − O1 − Mn	172.50°	174.33°	171.42°
Mn − O2–Mn	171.75°	173.50°	149.83°

*Values are taken from reference [28]

2 $${OS}_{\parallel }=\frac{2(c-a)}{(c+a)}$$

3 $${OS}_{\perp }=\frac{2(a+c-b\sqrt{2})}{(a+c+b\sqrt{2})}.$$

It can be seen in Table 2 that in the nanoparticles of average diameter 4 nm, the orthorhombic strain is highest and it also has severe reduction of one of the bond angle Mn-O2-Mn which suppresses the transfer integral and suppresses the ${T}_{C}$ [32].

The results are displayed in the graphs below where the variations of the important structural parameters as a function of the stoichiometry $\delta$ are shown.

**Fig. 3:**
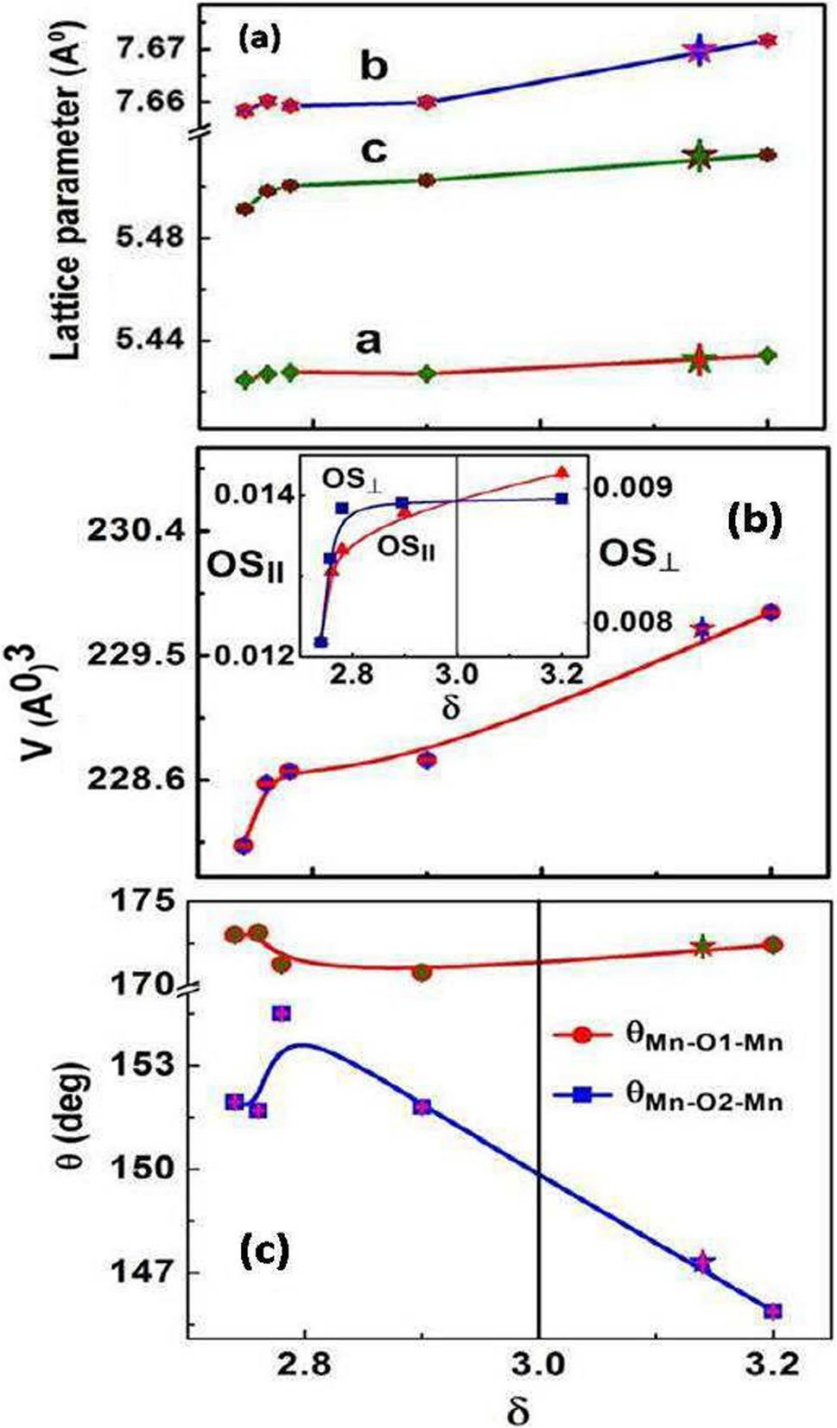
Oxygen stoichiometry (δ) dependence of (**a**) lattice parameters, (**b**) crystal volume and (**c**) bond angles are shown. Star (⋆) symbols are the results of reduced sample S^⋆^. Error bars are smaller than the symbols

The changes of lattice parameters for different values of $\delta$ are shown in Fig. 3a–c. At the initial stages of oxidation ($\delta$ changes from 2.74 to 2.76) lattice constants *a*, *b*, *c* and the unit cell volume *V* sharply increase. After that the values increase gradually with δ*.* The initial sharp increment is more prominent for *c* and *V*. For the change of $\delta {\text{on}}$ oxidation from 2.74 to 3.20 (= 0.46) the value of *V* changes from 228.2 (*A*^0^)^3^ to 229.8 (*A*^0^)^3^ (~ 0.7%). Results on the sample S^*^ which is reduced sample from S_5_, shows that the electrochemical process can be reversible at least within the limit for which it has been checked. The variations of orthorhombic strains ${OS}_{\parallel }$ and ${OS}_{\perp }$ with $\delta$ are shown in the inset of Fig. 3b. For the initial change of$\delta$, ${OS}_{\parallel }$ and ${OS}_{\perp }$ increase sharply and after that change in ${OS}_{\perp }$ saturates, although ${OS}_{\parallel }$ has a gradual increment. The particle size effect on the orthorhombic strain can be clearly visualized from Table 2. From Fig. 3c it can be seen that the bond angle Mn-O1-Mn remains close to the ideal value of 180^0^, which happens in a cubic structure with orthorhombic distortion $\to 0.$ However, as the orthorhombic distortion increases on oxidation, the bond angle Mn-O2-Mn decreases substantially after a small initial rise. The change in the bond angle on oxidation is an important parameter.

### Change in magnetic properties after electrochemical oxidation/reduction:

**Fig. 4:**
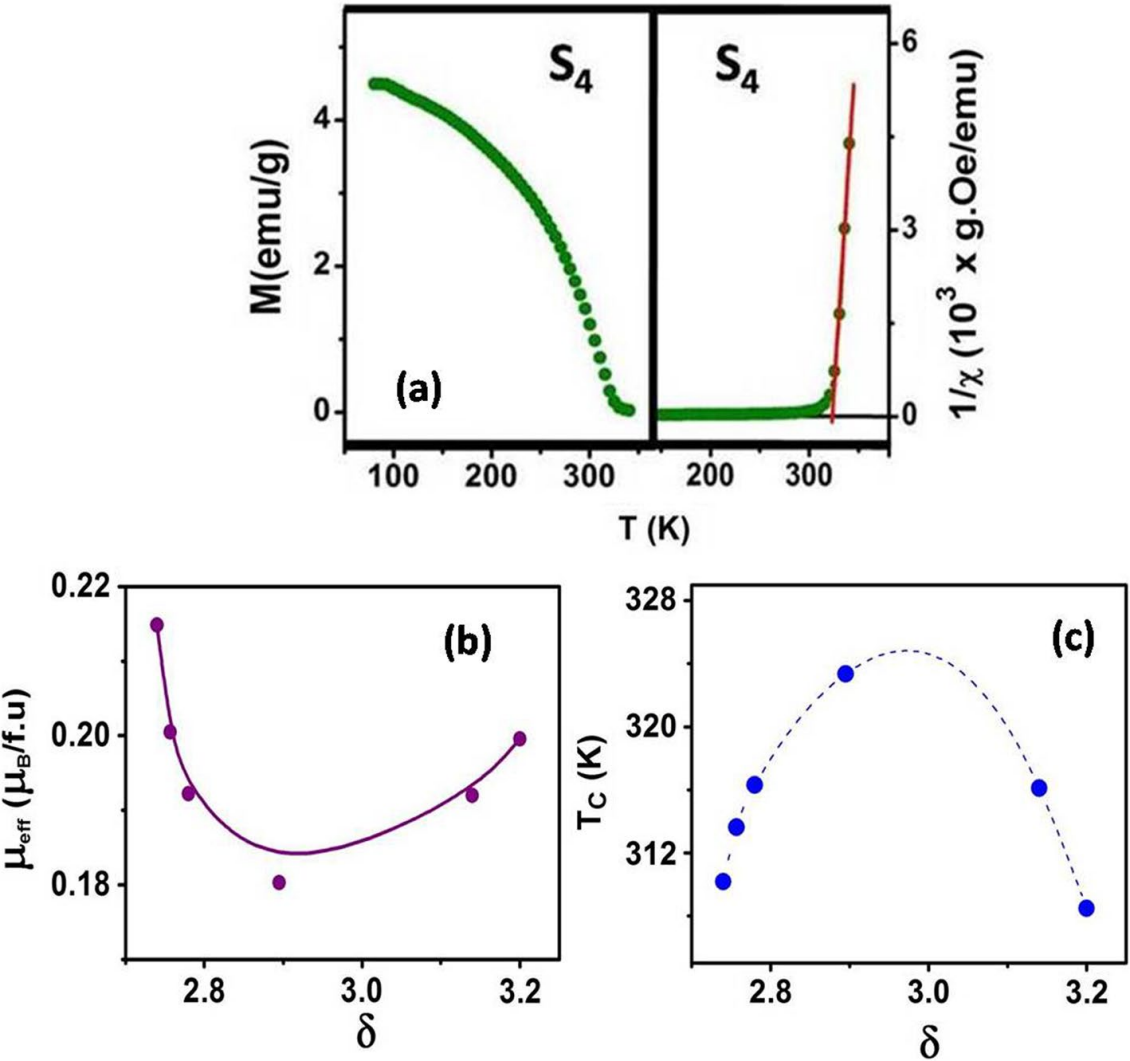
**a** Temperature dependence magnetization at a field of 0.01 T and *1/χ* vs. temperature fitting of *S*_*4*_ **b** Effective magnetic moment ${\mu }_{eff}$ per formula unit and **c** Curie temperature (*T*_*C*_) are plotted with respect to *δ*

The change in $\delta$ alter the magnetic properties like the Ferromagnetic Curie Temperature *T*_C_, effective moment ${\mu }_{eff}$, saturation magnetization ${M}_{S}$ and the coercive field *H*_C._ Development of the effective moment as a function of the stoichiometry $\delta$ shows that there is qualitative difference with the behaviors observed in the bulk [33]. We also observe large enhancement of the Curie Temperatures ${T}_{C}$ in nanoparticles with average diameter of 4 nm compared to those observed in the bulk. *T*_C_ and ${\mu }_{eff}$ have been obtained from the Curie–Weiss law *χ* = *C*/(*T − T*_C_), where *χ* is the susceptibility measured at a low field of ${\mu }_{0}H=$ 0.01 T in the paramagnetic region ${T>T}_{C}$. The effective magnetic moment ${\mu }_{eff}$ has been obtained from the relation $C=\frac{{\mu }_{eff}^{2}}{3{k}_{B}}$.The values of ${\mu }_{eff}$ and ${T}_{C}$ are tabulated in Table 3 and the two parameters are plotted as a function of $\delta$ in Fig. 4a and b.

**Table 3:** The values of ${\mu }_{eff },{T}_{C}$ and ${M}_{S}$ are tabulated below

Sample-Id	$\delta$	${\mu }_{eff}$(${\mu }_{B}$)	*T*_*C*_(K)	${M}_{S}$(${\mu }_{B}$/f.u)
*S*_*1*_	2.74	0.215 (± 0.006)	310.2 (± 1.2)	1.132 (± 0.008)
*S*_*2*_	2.76	0.200 (± 0.006)	313.6 (± 0.6)	0.983 (± 0.009)
*S*_*3*_	2.78	0.192 (± 0.004)	316.3 (± 0.4)	0.993 (± 0.009)
*S*_*4*_	2.90	0.180 (± 0.006)	323.4 (± 0.7)	0.833 (± 0.006)
*S*_*5*_	3.20	0.199 (± 0.003)	308.5 (± 0.4)	1.064 (± 0.007)
S*	3.14	0.191 (± 0.005)	316.1 (± 0.6)	0.893 (± 0.011)

The magnetization vs. temperature curve shows a ferromagnetic nature (shown in Fig. 4a), where the magnetization $M$ rises rapidly below the Curie temperature ${T}_{C}$. The Curie temperature *T*_*C*_ was determined from the linear fitting of the temperature dependent 1/χ plot. The Curie temperature shows a non-monotonous dependence on $\delta$ and expectedly reaches the highest *T*_C_ (324 K) for *δ* ≈ 3 and decreases by nearly 15 K when $\delta$ deviates from the optimum value 3 on either side (shown in Fig. 4c). The observed *T*_C_ of 324 K in nanoparticles of LCMO is the highest reported and is substantially larger than the bulk samples or single crystals and is even larger than *T*_C_ = 290 K observed in LCMO nanoparticles of diameter ~ 50 nm [28, 34, 35]. The enhanced *T*_C_ is comparable to 315 K observed in 65 nm LCMO nanowires grown in alumina templates [36].

We also observe that by electrochemical reduction (sample S^⋆^) it is possible to reverse the oxidation reproducibly thus establishing that the electrochemical route provides a reversible way to control the stoichiometry.

The effective moment ${\mu }_{eff}$ also shows a non-trivial dependence on $\delta$ as shown in Fig. 4b and Table 3. It shows a sharp drop when the manganite nanoparticles are oxidized till the optimum value of $\delta =3,$ beyond that ${\mu }_{eff}$ slowly recovers. The reduction in ${\mu }_{eff}$ on oxidation or hole doping (that can also be achieved by substitution in La site by di-valent Ca) has been observed in manganites in bulk form where ${\mu }_{eff}$ shows a peak at hole concentration less than that of optimum hole doping [37] and then gradually decrease mainly due to presence of more Mn^4+^ that has a lower spin value. The enhancement of ${\mu }_{eff}$ beyond the optimum hole doping as has been observed in nanoparticles have not been observed in the bulk form. This issue will be further discussed later on.

**Fig. 5:**
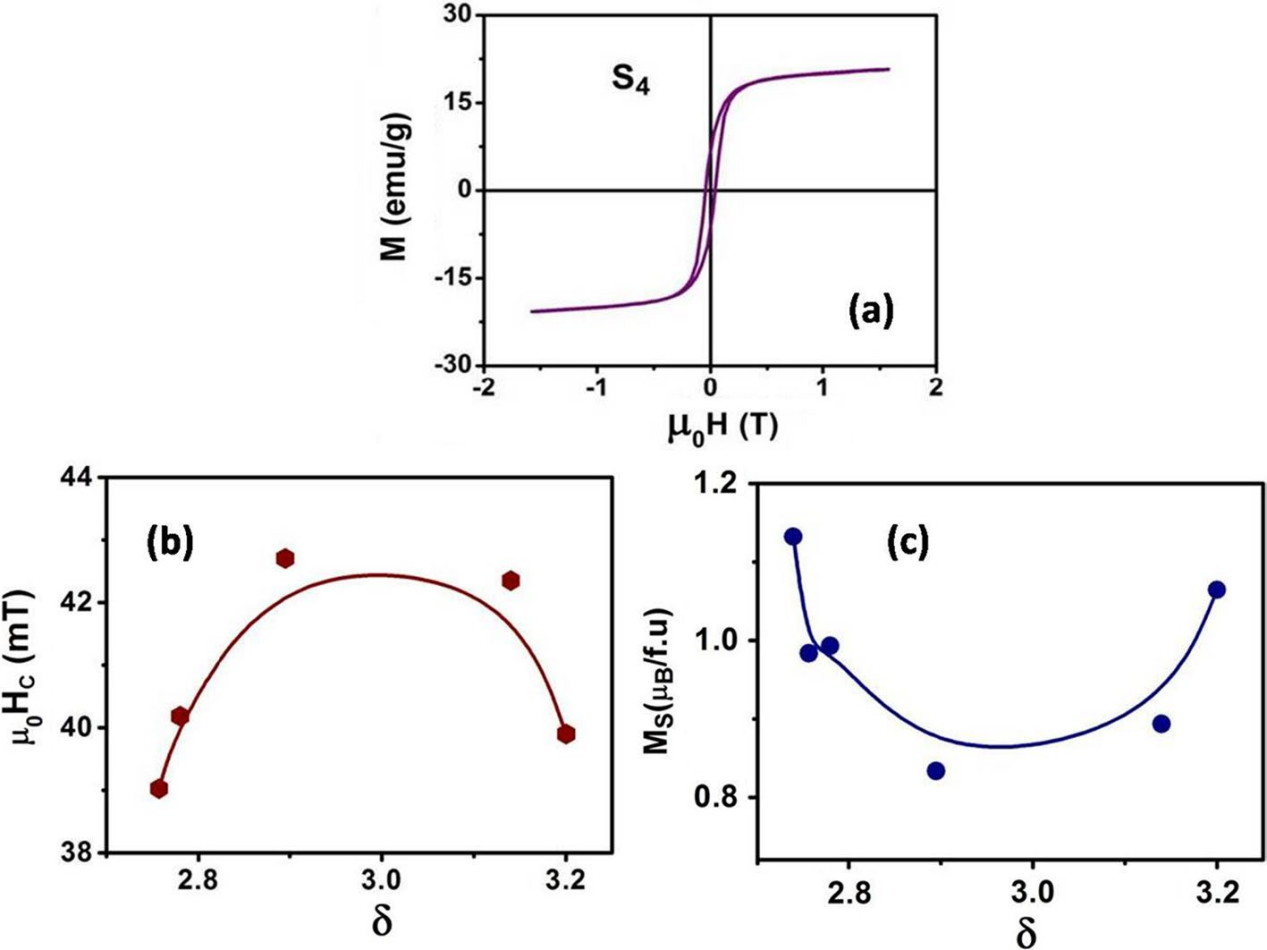
**a** Magnetization versus field curves of *S*_*4*_ at temperature 80 K **b** Coercive field and **c** saturation magnetization versus $\delta$ is plotted

The ferromagnetic nature of the sample is clearly visible in the field dependent magnetization curve (Fig. 5a) that shows hysteresis in the $M-H$ curve. In Fig. 5b, c and Table 3 we show the saturation magnetization *M*_S_ at 80 K along with the coercive field (*H*_C_). *H*_C_ shows a maxima close to the stoichiometry $\delta \approx 3$ and there is small decrease (~ 10%) when $\delta$ deviated from the optimum value. The ${M}_{S}$ shows a minima around $\delta \approx 3$ and follows the same trend as the magnetic moment ${\mu }_{eff}$. The ratio $\frac{{M}_{S}}{{\mu }_{eff}}$ remains almost constant to within $\approx \pm 7\%$ as $\delta$ is varied.

We find that the product ${M}_{S}{H}_{C}$, is nearly independent of the value of $\delta$ and it varies by <$\pm 5\%$ when $\delta$ is changed by more than 15%. In the size domain used by us the nanoparticles are monodomains. The size range where the nanoparticles of manganites become mondomain is $\approx$ 70–75 nm as determined by the exchange constant (0.3 eV) and the saturation magnetization. In absence of agglomeration, in such a system, near independence of the ${M}_{S}{H}_{C}$ product would imply constancy of the anisotropy constant $\kappa$ as the parameter $\delta$ is changed (the constant $\kappa$ is given related to ${H}_{C}$ through the relation ${H}_{C}\propto \frac{2\kappa }{{M}_{S}}$.) We however, note that the above relation may get modified by agglomeration and we exercise caution on the above inference. In the case that $\kappa$ is nearly independent of $\delta$, ${H}_{C}$ is tagged to ${M}_{S}$ which in turn is tagged to the magnetic moment ${\mu }_{eff}$ shows a minimum value around $\delta \approx 3$.

In optimally doped LCMO with *δ* ≈ 3 the value of ${M}_{S}$ at *T* = 80 K is around ≈ 3 $.42{\mu }_{B}$/Mn [38]. On size reduction the value of ${M}_{S}$ is suppressed and for LCMO nanoparticles with average diameter ~ 50 nm it is ≈ 1.$5{\mu }_{B}$/Mn [38] and in the size range of our sample (~ 4 nm) it is further reduced to only ≈ 1.$0{\mu }_{B}$/Mn. Similar trend is also seen in the values of magnetic moment ${\mu }_{eff}$. For LCMO with $\delta \approx 3$, the Mn^4+^ content is 33.3% while Mn^3+^ content is 67%. The expected spin only value of ${\mu }_{eff \approx } 4.56{\mu }_{B}$. For the nanoparticle ${\mu }_{eff}$ is much suppressed and is in the range of 0.2 ${\mu }_{B}$.

## Discussion

The process of oxidation changes the Mn $-$ O $-$ Mn bondangles can seen from Fig. 3c.While the bond angle Mn $-$ O1 $-$ Mn remains mainly unaltered, the angle Mn $-$ O2 $-$ Mn increases sharply on initial oxidation and then decreases. Since the increase of the Mn $-$ O $-$ Mn bond angle to 180^0^ will increase the orbital overlap, this will enhance the band width leading to enhancement of *T*_C_ which being a double exchange mechanism is directly related to the band-width. However, for $\delta$ > 2.9, the Mn $-$ O2 $-$ Mn angle decreases again but the enhancement of *T*_C_ continues still higher $\delta$ (~ 3) and then it decreases again. Thus the enhancement of *T*_C_ for $\delta$ ≤ 3 will not only depend on the change in the bond angle but also on other factors like the exchange constant. For $\delta$ > 3 the suppression of *T*_C_ is most likely is governed by the large decrease in the bond-angle Mn $-$ O2 $-$ Mn.

For nanoparticles, depression of ${M}_{S}$ from the bulk value can arise from surface oxides with lower oxygen content which will suppress their ferromagnetism. This acts as a “dead layer” on the surface of the nanoparticles. This layer would contribute to mass or volume but not to magnetic moment and as a result the effective magnetization/unit volume or unit mass will become smaller than the bulk value [28]. However, ${T}_{C}$ which is an intensive quantity, will not be affected by the “dead layer”. The particle size distribution shows that there is a finite fraction of the nanoparticles have diameter $\le$ 2 nm. For these particles, the magnetization will be suppressed and may even be paramagnetic. The volume of these nanopartciles will thus be counted in the volume of the “dead layer”.

For a nanoparticle of diameter $d$, if the surface “dead layer” has a thickness of $\lambda$ and is assumed to have a zero magnetization, the magnetization will be contained within the core of radius = (d/2-λ). If the magnetization of the nanoparticles is ${M}_{S}$, and that of the ideal nanoparticle with magnetization same as the bulk is ${M}_{0S}$, then the two are related by the relation:

4 $${M_S} = {M_{OS}}{\left( {\frac{{\frac{d}{2} - \lambda }}{\frac{d}{2}}} \right)^3}$$

For the optimally doped sample of LCMO with $\delta \approx$ 3,${M}_{OS}=$ 3.42 ${\mu }_{B}$ at 80 K [37]. From Eq. (4) the value of $\lambda$ can be calculated using the relation:

5 $$\lambda = \left\{ {1 - {{\left( {\frac{{M_S}}{{{M_{OS}}}}} \right)}^{{\raise0.7ex\hbox{$1$} \!\mathord{\left/ {\vphantom {1 3}}\right.\kern-0pt}\!\lower0.7ex\hbox{$3$}}}}} \right\}\frac{d}{2}$$

**Fig. 6:**
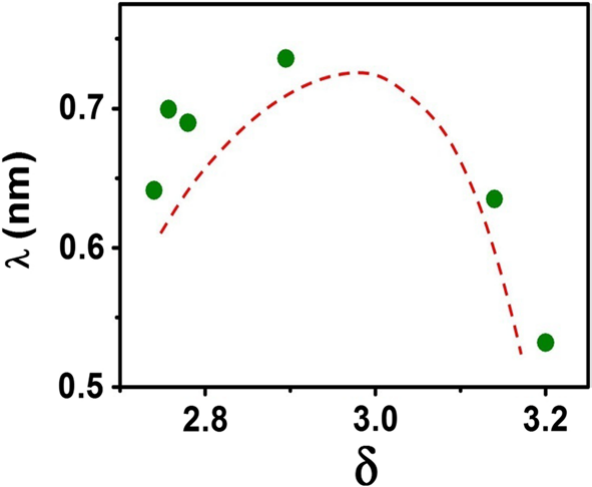
Variation of shell thickness (*λ*) versus $\delta$ plot

From Eq. (5) we evaluated the dead layer thickness (*λ*) as a function of $\delta$. This is shown in Fig. 6. There is a shallow enhancement of $\lambda$ for the initial stage of oxidation, $\lambda$ reaches a maximum at $\delta$≈ 3. For further increase of $\delta$ the value of $\lambda$ reduces sharply by 30% and leads to enhanced ${M}_{S}$ as shown in Fig. 5. The reduction of $\lambda$ for higher values of $\delta$ happens because the oxidation process increases the oxygen content in the dead layer as well and thus reduces the effective thickness of the dead layer.

## Summary

We investigated effect of change in Oxygen stoichiometry ($\delta$) in small nanoparticles of La_0.67_Ca_0.33_MnO_δ_ with average diameter ~ 4 nm. The stoichiometry $\delta$ was changed by electrochemical oxidation/reduction without changing the particle size, unlike heating based methods where change in $\delta$ also leads to change in particle size. The XRD data shows Orthorhombic strain becomes very large in such small particles. The change in $\delta$ leads to non-monotonous variation of the Mn-O2-Mn bond angle that modifies the physical properties including non-trivial change in ferromagnetism linked properties such as ${T}_{C}, {M}_{S}$ and ${H}_{C}$.Very small particle size makes it possible to carry out large change in oxidation from *δ* = 2.74 to *δ* = 3.20 due to faster diffusion.

It is an important observation that the ferromagnetism persists even down to such small sizes, although with strongly suppressed ${M}_{S}$. This suppression of ${M}_{S}$ is linked to existence of a magnetically dead layer on the surface. The process of electrochemical oxidation leads to reduction of thickness of the dead layer.

### Supplementary Information

**Additional file 1.**

## Data Availability

The datasets generated during and/or analysed during the current study are available from the corresponding author on reasonable request.
