# Supplementary material for: Nanoscopic oxygen control of functional oxide nanoparticles by electro-chemical route at ambient temperature
Source: Discov Nano. 2024 Feb 8;19(1):25. doi: 10.1186/s11671-024-03969-y (PMC10853112; doi:10.1186/s11671-024-03969-y)
Supplement: Supplementary file 1 — Additional file 1. [file 11671_2024_3969_MOESM1_ESM.docx]

NanoscopicOxygen controloffunctionaloxidenanoparticlesbyelectro-chemicalroute at ambient temperature

**Supplementary information:**


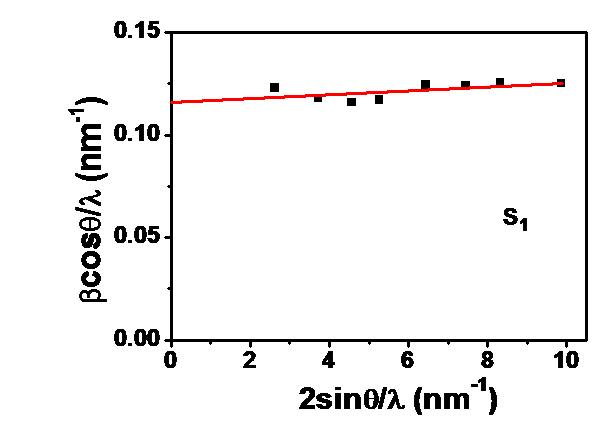


**FIG. S1.** Williamson Hall plot from XRD results of sample S_1_.

The particle size ($d$) and micro strain ($\varepsilon$) of the system is related with the Full width half maximum ($\beta$) of XRD peaks as,

$$\frac{\beta cos\theta}{\lambda}=\frac{1}{d}+\varepsilon\frac{2sin\theta}{\lambda} (S1)$$

, where $\lambda$= 0.1518 nm is the wave length of Cu Kα and *θ* is the XRD peak angle for a given Miller indices (hkl). From Williamson-Hall plot (FIG. S1) we get the average particle size ~ 8 nm of the sample S_1_.
